# Destructive Xylazine Wounds: A New Entity Faced by Thoracic Surgeons

**DOI:** 10.1016/j.atssr.2025.01.011

**Published:** 2025-02-13

**Authors:** Louis F. Chai, Whitney M. Burrows, Joseph S. Friedberg, Charles T. Bakhos, Lisa Rae, Cherie P. Erkmen

**Affiliations:** 1 Department of Thoracic Medicine and Surgery, Temple University Hospital, Philadelphia, Pennsylvania; 2 Division of Burn, Trauma, and Surgical Critical Care, Department of Surgery, Temple University Hospital, Philadelphia, Pennsylvania

## Abstract

Xylazine has caused increasing drug-related mortality and necrotic wounds. Neck and chest injections have caused destructive lesions that have engaged thoracic surgeons. We present 3 cases from Philadelphia, the epicenter of xylazine use, and discuss the multidisciplinary care effort. Providers should be aware of the symptoms of xylazine use to appropriately treat patients.

Wounds and infections from drug injections occur commonly in persons who uses drugs, typically in the extremities. Severity ranges from cellulitis to necrotizing fasciitis. Management is supportive with antibiotics and local wound care with surgery reserved for severe infections.

Recently, xylazine has become popularized, with Philadelphia as an epicenter of use.^1^ It is used increasingly with fentanyl, where an estimated 90%+ of samples contain xylazine. Drug-related deaths have increased 1238% nationwide, causing a public health emergency.^1^^,^^2^ As a central alpha-2 agonist, xylazine inhibits catecholamine release, resulting in sedation, analgesia, and euphoria. Fentanyl combinations amplifies this and increases effect duration.^2^

Xylazine injections have also led to a new wound phenotype. Whereas previous infections were generally minor, xylazine wounds are characterized by necrosis with significant tissue loss. As extremity venous access becomes limited from repeated injections, alternative routes are used, such as subcutaneous. This can continue in the extremities but have become increasingly central, resulting in neck and chest wounds. Temple University is centered in Philadelphia, where there is the highest recorded incidence of xylazine use. Numerous patients with extremity wounds receive care here, but these new destructive chest wounds have engaged the thoracic surgeon. We report a series of these injuries and the multidisciplinary management algorithm.

## Case Reports

### Patient 1

**Figure 1:**
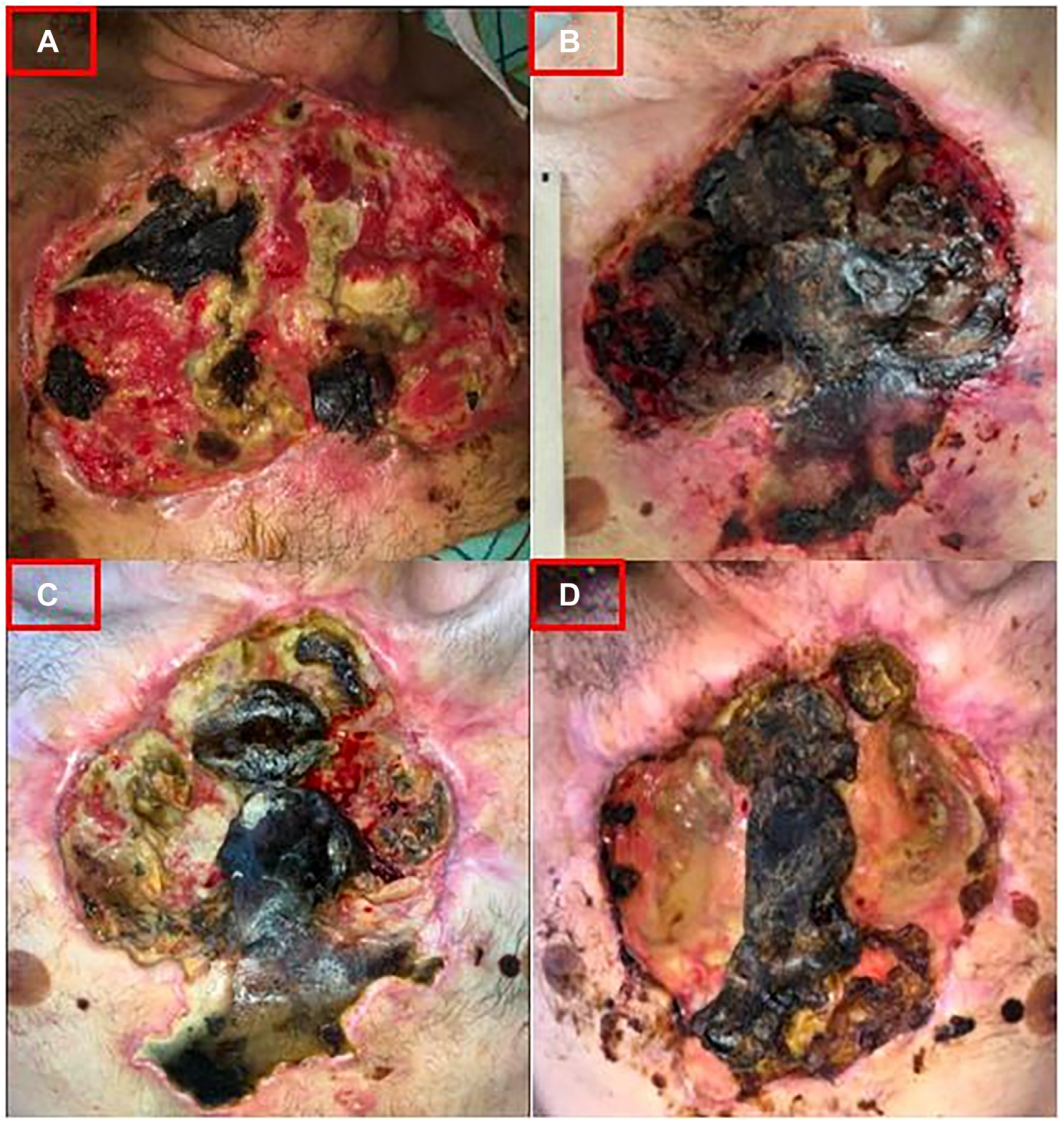
Interval images of patient 1. (A) Initial presentation. (B) Eight-month interval. (C) One month after daily wound care. (D) Final visit.

A 32-year-old male patient presented for chest ulceration and imaging showed considerable soft tissue defects (Figure 1A). Debridement was performed with more invasive intervention planned, but the patient left via patient-directed discharge (PDD), returning 2 months later after continued injections, with worsening necrosis and exposed bones. Given the complex surgery, infection, and psychosocial barriers to care, thoracic, burn, and plastic surgery, addiction medicine, infectious disease, and social work convened for care planning. One concern was his ongoing drug use and assessing the risk of extensive surgery where success depended on reliable perioperative care. The team planned for prolonged antibiotics and detoxification in a facility prior to surgery. However, while hospitalized, the patient self-injected xylazine and left via PDD.

**Supplementary Figure 1:**
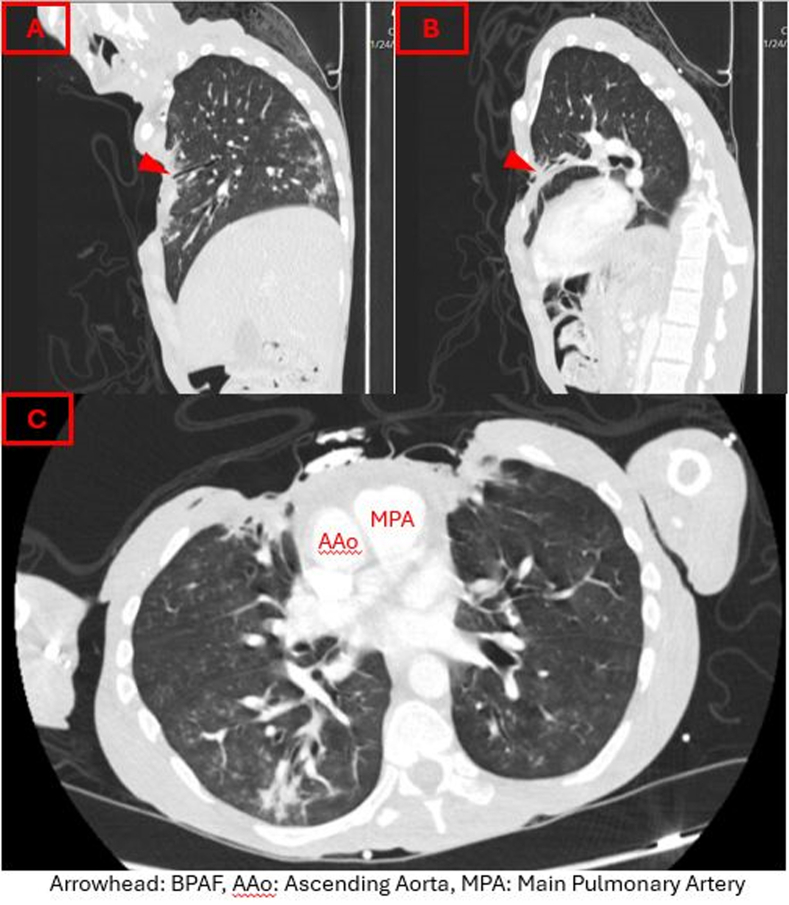


He had inconsistent care over the following 6 months, but when wound bubbling developed concerning for broncho-pleural-atmospheric fistula (BPAF), he was readmitted (Figure 1B). Imaging showed minimal soft tissue, sternal destruction, BPAF, and lung infarction. Psychiatry and palliative care became involved, determining consent capacity for radical surgery despite prior self-harm behavior. He received daily wound care while planning intervention with significant improvement (Figure 1C). However, the patient again self-injected xylazine and left via PDD after 1 month. He had 3 subsequent emergency department visits but left as a PDD each time. His wound remains completely necrotic with BPAFs and minimal tissue coverage of underlying structures (Figure 1D, Supplemental Figures 1A-1C).

### Patient 2

**Figure 2:**
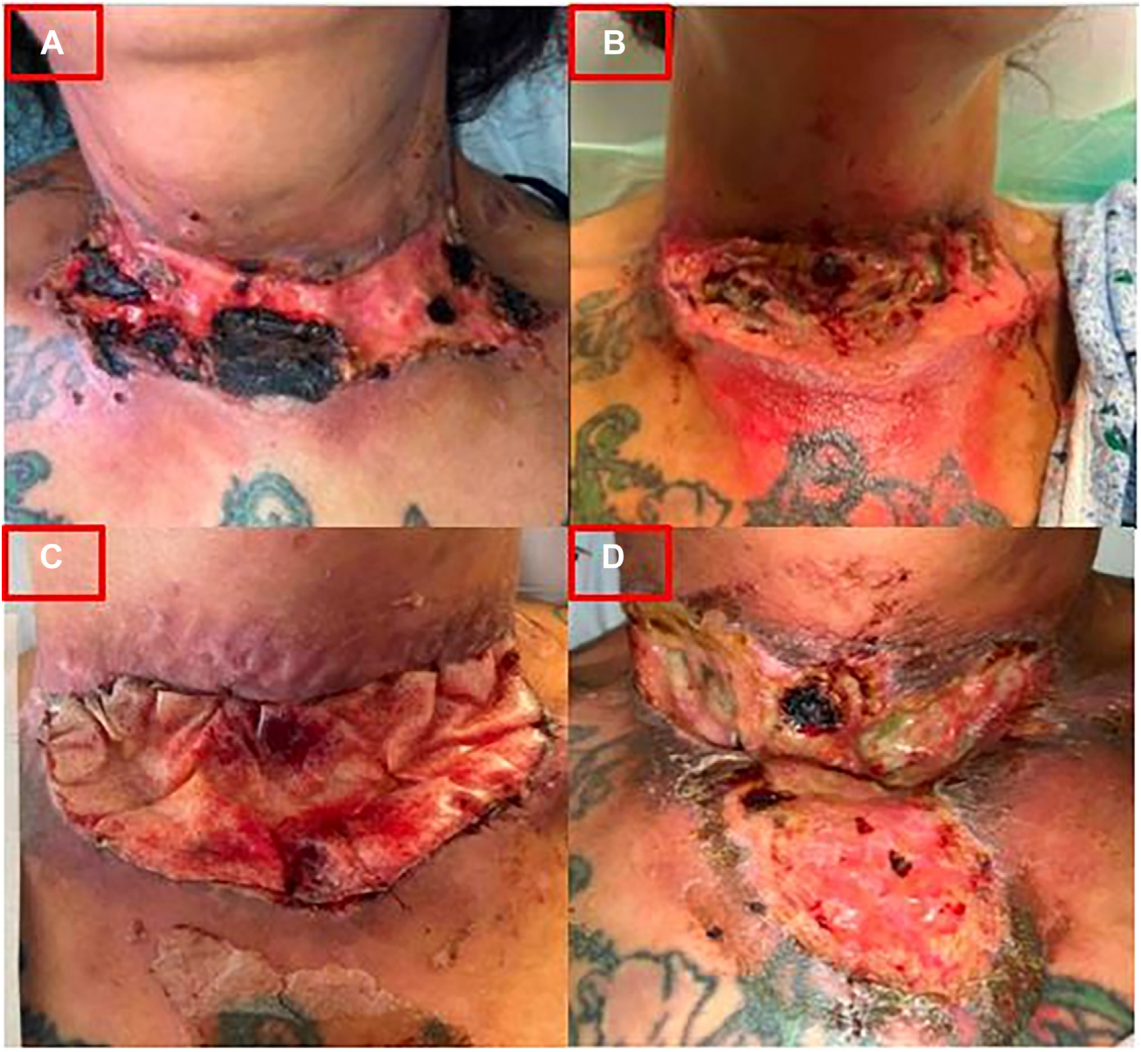
Interval images of patient 2. (A) Initial presentation. (B) Three-week interval. (C) Three-month interval, post-debridement with dermal substitute. (D) Five-month interval.

A 32-year-old female patient presented with 4 months of a superficial, necrotic neck wound with continued injections (Figure 2A). Local wound care and antibiotics were recommended but she left via PDD. Three weeks later, she returned with an enlarging, erythematous, but still superficial wound (Figure 2B). Again, local wound care and antibiotics were recommended on discharge.

Two months later, the patient was readmitted with exposed musculature and she was debrided with dermal substitute placement (Figure 2C). More intervention was planned once appropriate care was established, but the patient left via PDD again before readmission 2 months later with necrosis, increased wound size, and polymicrobial infection (Figure 2D). She underwent local debridement and tissue substitute placement with repeated intervention planned, but again left via PDD and has since been lost to follow-up.

### Patient 3

**Figure 3:**
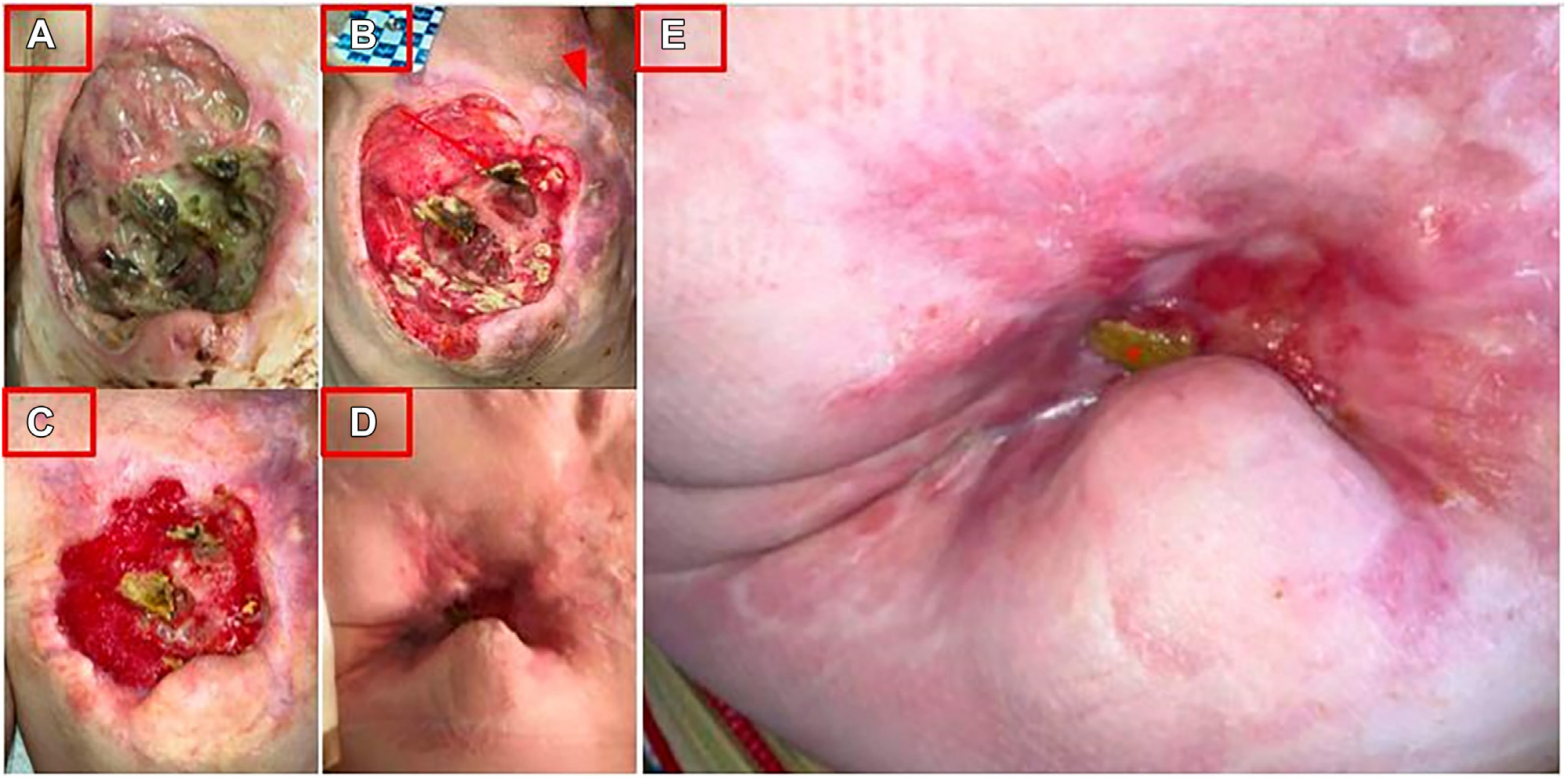
Interval images of patient 3. (A) Initial presentation. (B) One-week interval. (C) One-month interval. (D) Six-month interval. (E) Nine-month interval. The arrowhead shows the sternoclavicular joint.

**Supplementary Figure 2:**
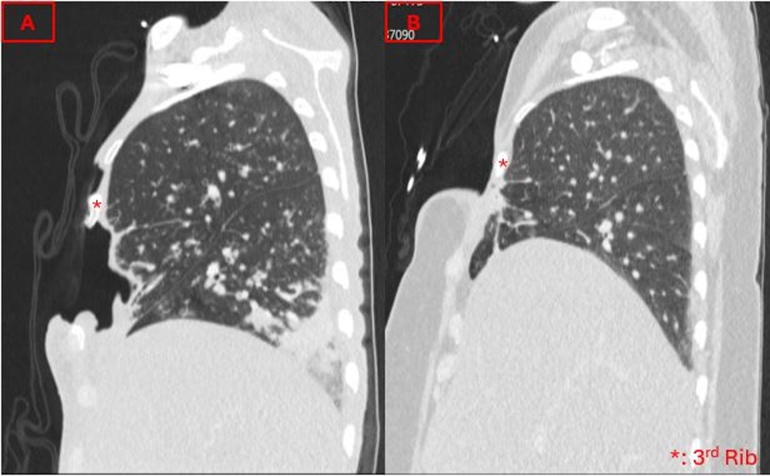


A 32-year-old female patient presented with worsening chest and lower extremity wounds, tachycardia, and acidosis. Chest examination showed exposed ribs and BPAF (Figure 3A). Imaging showed preserved lung and no abscesses (Supplemental Figure 2A). She notably had a lower extremity necrotizing infection, requiring amputation. Her chest wound was managed conservatively given no undrained collection or uncontrolled BPAF.

The patient recovered over 2 months, receiving daily chest wound care, antibiotics, and worked extensively with addiction medicine (Figures 3B, 3C). She was ultimately discharged and 6 months later, and remained sober and socially supported. There was a small residual defect with reported bubbling, though there was no visualized BPAF (Figures 3D, 3E). Imaging showed minimal tissue deficit, but obvious rib destruction (Supplemental Figures 2A, 2B). She is planned for surgery for the remaining defect.

## Comment

Xylazine injections have resulted in large, necrotic wounds. The mechanism is related to alpha-adrenergic properties causing vasoconstriction and ischemia.^2^^,^^3^ The wound enlarges due to poor healing, repeated injections, and/or infections. The tissue loss on presentation is attributable to unawareness of severity or lack of pain due to analgesia. Previously localized to the extremities, new devastating wounds of the thorax emerged as alternative injections sites are used.

Xylazine wound management is limited to case reports.^1^^,^^4^, ^5^, ^6^, ^7^ Care relies on debridement and local wound care. Most wounds heal by secondary intention, though larger wounds or areas with exposed bones, tendons, or vasculature may require tissue or dermal substitute coverage. These same principles apply to neck and chest wounds. Superficial wounds can be debrided with additional tissue coverage followed by local wound care. One prior report corroborates this with good results.^7^ Even cases of notable defects or BPAFs do not necessitate emergent intervention in the absence of instability or undrained collections. Local wound care promotes tissue growth to close BPAFs and cover exposed structures. Radical interventions are morbid and intervening in the acute phase may be detrimental. Surgery is considered when conservative measures fail, for residual deficits, or as salvage.

A multidisciplinary effort is required for management. Ensuring adequate cardiopulmonary physiology due to chest wall compromise is critical. Infectious disease is involved given high risk for resistant organisms. Finally, a multispecialty surgical team is required for perioperative management, surgery, and wound care. From a clinical standpoint, these patients are remarkably stable despite severe wounds or BPAF. Except for patient 3, with necrotizing fasciitis, the patients were without metabolic derangements, suggesting that xylazine injuries are local processes. This further supports that such wounds are not surgical emergencies.

**Supplementary Figure 3:**
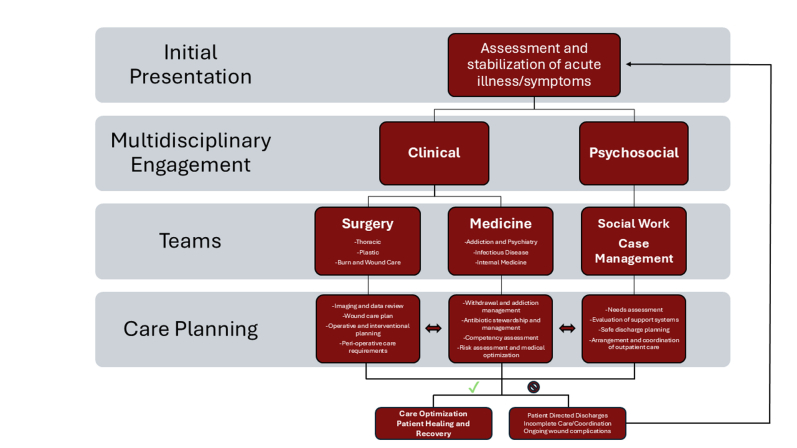


To that end, wound care represents only a small fraction of care. Management must include social work, addiction medicine, and psychiatry. This cohort faces issues related to homelessness, addiction, lack of support or healthcare access and/or transportation and financial limitations. These psychosocial barriers prevent patients from obtaining care, leading to PDDs, prolonged intervals between presentations, or self-harm behaviors—subsequently leading to potential disease progression. Thus, a comprehensive care team should incorporate clinical and care support disciplines (Supplemental Figure 3).

Xylazine use increases drug-related mortality rates.^3^ In addition to wounds, xylazine causes hypotension, arrhythmias, respiratory depression, and neurologic deficits. Early recognition of xylazine use is critical in treating persons who uses drugs. This series highlights a novel injury pattern to the chest and the multidisciplinary effort to manage such patients. Further understanding of xylazine-related pathologies will, we hope, result in more successful outcomes.

In conclusion, xylazine use is increasing and associated with necrotic wounds. Central injections result in destructive thoracic injuries, requiring multidisciplinary care to treat clinical and psychosocial variables.

## References

[1] McFadden R., Wallace-Keeshen S., Straub K.P. (2024). Xylazine-associated wounds: clinical experience from a low-barrier wound care clinic in Philadelphia. J Addict Med.

[2] Gupta R., Holtgrave D.R., Ashburn M.A. (2024). Xylazine—medical and public health imperatives. N Engl J Med.

[3] Bishnoi A., Singh V., Khanna U. (2023). Skin ulcerations caused by xylazine: a lesser-known entity. J Am Acad Dermatol.

[4] Malayala S.V., Papudesi B.N., Bobb R. (2022). Xylazine-induced skin ulcers in a person who injects drugs in Philadelphia, Pennsylvania, USA. Cureus.

[5] Warp P.V., Hauschild M., Serota D.P. (2024). A confirmed case of xylazine-induced skin ulcers in a person who injects drugs in Miami, Florida, USA. Harm Reduct J.

[6] Dowton A., Doernberg M., Heiman E. (2023). Recognition and treatment of wounds in persons using xylazine: a case report from New Haven, Connecticut. J Addict Med.

[7] O’Neil J., Kovach S. (2023). Xylazine-associated skin injury. N Engl J Med.

